# Integration of oral health into primary health care in Somalia: opportunities, barriers, and system-level implications for universal health coverage

**DOI:** 10.3389/froh.2026.1930618

**Published:** 2026-10-07

**Authors:** Saido Gedi, Salma Ali Ossoble, Mohamoud M. Dahir, Nasra Abdulsamad Mohamud, Abubakar Ahmed, Jihaan Hassan, Maye Abu Omar

**Affiliations:** 1 Faculty of Dentistry, Mogadishu University, Mogadishu, Somalia; 2 Ministry of Health and Human Services, Mogadishu, Somalia; 3 SIMAD Institute for Global Health, SIMAD University, Mogadishu, Somalia; 4 School of Medicine, Leeds Institute of Health Sciences, University of Leeds, Leeds, United Kingdom

**Keywords:** Essential Package of Health Services, fragile settings, health systems, noncommunicable diseases, oral health, policy implementation, primary health care, qualitative research

## Abstract

**Background:**

Oral diseases are among the most common noncommunicable conditions globally, yet oral health remains poorly integrated into primary health care (PHC) and universal health coverage, especially in fragile, low-limited settings. As Somalia rebuilds its health system through the Health Sector Strategic Plan III 2022–2026, the Essential Package of Health Services 2020, the Investment Case for the Somali Health Sector 2022–2027, and community health reforms, this study examined the place of oral health within national health policies. It identified barriers to integration and explored feasible pathways for strengthening PHC-based oral health.

**Methods:**

The study used a qualitative policy and health-systems analysis combining documentary review with 13 key-informant interviews. Participants represented national policymakers, health-system technical advisers, regulators, academics, professional associations, and providers at public hospital, and private dental settings. Data were analyzed thematically and mapped against the WHO health-system building blocks, PHC principles, and the WHO Global Strategy and Action Plan on Oral Health 2023–2030.

**Results:**

Somalia's policy environment offers opportunities for oral health integration, but stakeholders highlighted a persistent gap between policy recognition and implementation within routine PHC. Seven interrelated themes were identified: (1) policy recognition without operationalization; (2) fragmented governance, weak regulation, and limited coordination; (3) reliance on out-of-pocket financing and private-sector; (4) workforce, education, and task-sharing constraints; (5) predominantly curative, urban, hospital-based service with weak linkage to maternal and child health (MCH) and PHC; (6) limited oral health data and evidence generation; and (7) opportunities for phased integration through PHC, MCH, school health, community health workers, and NCD platforms Policymakers emphasized the need for stronger policy implementation, financing, regulation, standards, and data systems, while service providers highlighted unaffordable care, delayed care-seeking, workforce and infrastructure shortages, and missed opportunities for prevention.

**Conclusion:**

Oral health integration in Somalia is a broader health system challenge rather than solely a dental-service issue. The most feasible approach is a phased, system-based model that prioritizes strengthened governance, a defined minimum oral health within PHC, workforce task-sharing, screening, counselling, referral pathways, private-sector regulation, essential commodities, and oral health indicators in District Health Information System 2 (DHIS2), followed by the gradual expansion of curative and rehabilitative services.

## Introduction

Oral diseases are a major public health challenge worldwide, yet one that remains largely neglected*.* Conditions such as dental caries, periodontal disease, tooth loss, oral cancers, and oral manifestations of systemic disease affect individuals throughout the life course and disproportionately burden disadvantaged populations based on socioeconomic status, geography, education, gender, and access to care. Although largely preventable, Oral diseases affect an estimated 3.5 billion people worldwide, making them the most prevalent noncommunicable conditions globally; untreated dental caries of permanent teeth alone affects close to 2 billion people, and caries of primary teeth affects a further 514 million children (1, 2). This burden falls disproportionately on low- and middle-income countries (LMICs), where preventive services are least available, oral health workforce shortages are most acute, and reliance on out-of-pocket payment is highest (1, 2).

Addressing this burden requires embedding oral health within the broader global health policy agenda*.* The global policy landscape increasingly recognizes oral health as a core component of Universal Health Coverage (UHC), primary health care (PHC), and the broader noncommunicable disease (NCD) agenda (2). The World Health Organization's Global Strategy and Action Plan on Oral Health advocates a shift away from stand-alone, treatment-focused dental services toward integrated, prevention-focused, and people-centred models delivered through PHC (3). This approach emphasizes strengthened governance, incorporating essential oral health services into health benefit packages, developing the workforce, addressing shared NCD risk-factors ensuring access to essential medicines and technologies, and integrating oral health into routine health information systems. Consequently, in fragile and resource-constrained settings, oral health integration is not merely a service-delivery reform but a health system strengthening strategy that can improve access, promote equity, and reduce financial barriers by leveraging existing PHC platforms (4). Somalia provides an important context for examining oral health integration within PHC. Decades of conflict, institutional fragmentation, recurrent humanitarian crises, population displacement, and limited public financing have shaped the country's health system. Recent reforms, including efforts to strengthen PHC, implement the Essential Package of Health Services (EPHS), and improve coordination across federal and state levels, have created opportunities for health-system development. However, service availability remains uneven, donor dependence is substantial, the private sector plays a dominant yet variably regulated role, and out-of-pocket spending continues to be a major source of health financing (5). Within this context, oral health has received limited policy and programmatic attention. Dental care is largely reactive rather than preventive, with treatment often sought only when pain, infection, trauma, or advanced disease occurs. Services are concentrated in urban private clinics and hospitals, while rural populations, internally displaced persons, nomadic communities, women, children, older adults, and low-income households face significant financial and geographic barriers to accessing care (6). Compounding these barriers, the oral health workforce is severely constrained: Somalia has fewer than 100 practising dentists nationally and an acute shortage of dental specialists, with dental care delivered almost entirely through private, donor-supported, or university-affiliated facilities rather than the public sector (7). The marginalization of oral health in Somalia reflects wider challenges in health system governance, financing, workforce capacity, and service-delivery. Public facilities often lack dedicated dental units, trained personnel, essential equipment and supplies, infection prevention infrastructure, and clear referral pathways (7). Where services are available, they are largely limited to emergency and curative care particularly extractions and pain management with little emphasis on prevention, health promotion, early detection, fluoride use, school-based programmes, antenatal oral health counselling, tobacco and khat-related risk reduction, or integration with NCD and child health services. Financing arrangements remain weak, with most dental care funded through out-of-pocket payments and limited financial protection for patients. Moreover, donor-supported PHC initiatives have traditionally focused on communicable diseases, maternal and child health, nutrition, immunization, and humanitarian priorities, leaving oral health largely excluded from routine planning, budgeting, procurement, supervision, and information-systems (8).

While broader health-sector reforms emphasize PHC expansion, UHC, regulation, financing reform, and health-system strengthening, oral health has yet to be fully integrated into these agendas as a distinct and coordinated component of care. As a result, a significant policy–implementation gap persists: oral health is acknowledged within national service packages but remains weakly institutionalized across governance, financing, workforce planning, and PHC service delivery systems.

Current national policy instruments provide a foundation for oral health integration, but this remains limited and insufficiently operationalized. Somalia's Essential Package of Health Services (EPHS) 2020 includes selected oral-health-related interventions, such as oral health promotion, referral of dental conditions, dental extraction, drainage of dental abscesses, and management of dental emergencies. However, these provisions are narrowly focused on clinical care and are not supported by a comprehensive implementation framework defining service standards, workforce competencies, essential commodities, financing mechanisms, referral pathways, quality assurance processes, or routine monitoring indicators (9). More broadly, while health-sector reform emphasizes PHC expansion, UHC, regulation, financing reform, and health-system strengthening, oral health has yet to be fully integrated into these agendas as a distinct and coordinated component of care. As a result, a significant policy–implementation gap persists: oral health is acknowledged within national service packages but remains weakly institutionalized across governance structures, financing, workforce planning, and PHC service delivery systems (10). At the same time, Somalia has important opportunities to advance oral health integration. Existing PHC facilities, maternal and child health services, schools, community health workers, female health workers (11), outreach platforms, and health promotion structures could be leveraged to deliver oral health education, risk assessment, early detection, referral, and basic preventive interventions (12) Integration with existing programmes for NCD, nutrition, tobacco and khat cessation, antenatal care, child health, school health, and emergency services offers practical entry points that do not require the immediate development of a fully specialized public dental system (13).

Despite these opportunities, limited evidence exists on how oral health is currently positioned within Somalia's health policy framework, the system-level barriers that hinder its integration, and the actors and delivery platforms that could support implementation pathways for integrating oral health into PHC within a fragile and resource-constrained health system. In particular, no prior study has systematically combined national policy analysis with the perspectives of both system-level and service-delivery stakeholders to translate this recognized policy opportunity into an actionable integration pathway. This study therefore examines the positioning of oral health within Somalia's PHC and UHC agenda, identifies barriers and opportunities across key health-system domains, and develops an implementation-oriented framework for integrating essential oral health services into PHC. By moving beyond a descriptive assessment toward feasible implementation pathways, the study aims to inform Somalia's health reforms while generating transferable lessons for oral health integration and health systems strengthening in other fragile and resource-constrained settings.

## Methods

### Study design

This study employed a qualitative policy and health-systems analysis design that combined a review of relevant policy and technical documents with key-informant interviews. The study was designed to generate implementation-oriented evidence on integrating oral health into primary health care (PHC) and universal health coverage (UHC) reforms in Somalia, rather than solely describing the availability of oral health services.

The study was informed by the World Health Organization (WHO) Health System Building Blocks framework, PHC principles, and the WHO global oral-health agenda. Reporting adhered to established qualitative research standards commonly required by public health journals, including transparent documentation of participant characteristics, data sources, analytic frameworks, coding procedures, and measures undertaken to ensure rigor and trustworthiness (14, 15). A completed COREQ checklist and the interview guide are provided as Supplementary Materials.

### Policy and document review

National and international policy and technical documents relevant to oral health, PHC, UHC, and health-system reform were reviewed. Documents were identified through the Ministry of Health and WHO websites and consultation with key stakeholders. The document search and review were conducted in January-March 2026. A total of nine core policy and technical documents, published or issued between 2014 and 2026, met the eligibility criteria and were included in the review (Supplementary Table S1). Documents were eligible for inclusion if they: (i) addressed Somalia's health-system priorities or reforms; (ii) contained provisions relevant to oral health, PHC, UHC, health financing, workforce development, or service delivery; or (iii) provided international guidance relevant to integrating oral health into PHC. Documents that did not contain substantive information related to the study objectives were excluded.

Key documents Included documents comprised six national policy and strategic instruments Somalia's Health Sector Strategic Plan III (HSSP III) 2022–2026, the Essential Package of Health Services (EPHS) 2020, the Investment Case for the Somali Health Sector 2022–2027, the Somali Community Health Strategy (2015), The “Marwo Caafimaad” Programme: Compendium to implement Community-based Female Health Workers' Programme (2014), Somalia Community Health Strategy 2023–2027, together with three international technical instruments: the WHO Oral Health Country Profile: Somalia (2022), the WHO Global Oral Health Status Report (2022), and the WHO Global Strategy and Action Plan on Oral Health 2023–2030 (2024).

Documents were analysed using a directed, framework-based document analysis. Each document was read in full, and content relevant to oral health, PHC, and UHC was extracted into a structured matrix organized around the WHO health-system building blocks (leadership/governance, health financing, health workforce, service delivery, health information systems, and medical products/technologies) the same framework used to structure deductive coding of the interview data, allowing document-derived content and interview themes to be triangulated directly. Extracted content was used to determine how oral health was positioned within national policies and reform priorities; identify provisions concerning governance, financing, workforce, service readiness, essential commodities, information systems, and referral arrangements; and assess alignment with international recommendations for integrating oral health into PHC and UHC. Findings from the document review are summarized in Table 1 and were triangulated with the seven qualitative themes (Table 3) to develop the integrated policy analysis presented in the Results and the phased implementation pathway proposed in the Discussion.

**Table 1:** Policy and service-readiness context for oral health integration in Somalia.

Domain	Current position	Integration implication
HSSP III/EPHS/Investment Case	Integrated PHC, equity, UHC, phased implementation, and partner alignment are national priorities. (9, 10, 18)	Oral health can be positioned within existing reform platforms rather than as a vertical dental programme.
WHO Somalia Oral Health Country Profile	High estimated burden of untreated caries, periodontal disease, and oral cancer; no national oral health policy/action plan reported in 2021. (19)	A policy roadmap and minimum oral health package are needed.
Public PHC service readiness	Oral health screening, urgent oral care/pain relief, and basic restorative care were not available in public PHC facilities in 2021. (9)	Integration should begin with screening, counselling, pain/infection triage, referral, and selected emergency care.
Mixed public-private health system	Dental care is concentrated in private and selected urban facilities, with weak regulatory and reporting systems. (7)	Licensing, accreditation, referral agreements, infection-prevention standards, and DHIS2 reporting are essential.
Community health reform	Somalia is strengthening community health and PHC resilience. (10)	CHWs/Marwo Caafimaad, school health, nutrition, ANC, immunization, and NCD platforms offer practical entry points.

Subthemes are mapped according to the World Health Organization (2007) Health Systems Framework building blocks, Theme 7 is mapped to “All building blocks” because its subthemes span leadership/governance (national roadmap, coordination), health workforce (CHW/nurse/midwife training), service delivery (referral protocols, school/MCH platform integration), health financing (phased financing and commodity readiness), and health information systems (pilot DHIS2 indicators); it was treated analytically as the integrative pathway drawing together the preceding six themes rather than as a single-building-block theme.

### Study setting and participant recruitment

Key informant interviews were conducted in Mogadishu between March and June, 2026. Participants were selected through purposive, criterion-based sampling to capture perspectives from institutions and professional groups directly involved in oral-health policy, regulation, education, planning, financing, or service delivery in Somalia.

Eligible participants were required to (i) hold a policy, regulatory, technical, academic, professional-association, managerial, or clinical role relevant to oral health or health-system development; (ii) possess direct knowledge or experience of Somalia's oral-health services or PHC reforms; and (iii) be willing to provide informed consent. Potential participants were identified through oral health focal person at the ministry, Somali Dental professional associations and contacted through telephone and email. Recruitment sought institutional and functional diversity rather than statistical representation.

A sample of thirteen key informants was considered appropriate given the relatively small and identifiable population of senior stakeholders occupying leadership, technical, regulatory, and clinical roles relevant to oral health policy and system-level service delivery in Somalia; this sample encompassed representatives from each major stakeholder category relevant to the study's objectives. Recruitment and data collection continued iteratively, with preliminary coding conducted alongside ongoing interviews. Saturation was assessed both overall and within each of the three broad stakeholder categories represented in the sample (policy and system-level actors; academic and professional-association representatives; and service-delivery participants): interviews within each category continued until no substantively new codes or subthemes relevant to that group's perspective emerged from successive transcripts. Because each stakeholder category comprised a small and well-defined population of senior, identifiable individuals in Somalia (Table 2), saturation within groups was further corroborated by the consistent convergence of policy-level and service-delivery accounts on the same seven themes (Table 5), rather than relying on numerical repetition alone.

**Table 2:** Participant characteristics and analytic grouping.

ID	Analytic group	Dominant role	Setting	Experience	Main interview focus
PM01	Policymaker	Federal Ministry of Health oral health lead	National oral health unit/PHC policy support	5 years	Policy, EPHS implementation, preventive programming
PM02	Technical partner	WHO focal point for health systems strengthening and global health initiatives	Health systems/PHC strengthening	>13 years	EPHS, PHC, financing, workforce, HIS
PM03	Technical advisor	Strategic technical advisor to Ministry of Health	National strategies, guidelines, roadmaps, SOPs	>20 years	Policy implementation, BPOC, DHIS2, financing
PM04	Regulator	Secretary General, National Health Professional Council	Licensing, accreditation, competency standards	1 year in role	Regulation, workforce standards, accreditation
PM05	Academic/policy leader	Dean of Faculty of Dentistry and senior dental officer	Dental education, EPHS, community advocacy	10+ years	Policy prioritization, workforce, stepped-care model
AC01	Academic leader	Dean, Faculty of Dentistry	Curriculum, policy dialogue, workforce training	4 years	Dental education, evidence generation, PHC integration
AC02	Academic coordinator	Faculty academic coordinator	Curriculum monitoring, teaching supervision, community programs	2 years	Curriculum, research gaps, task-sharing
PA01	Professional association/clinical leader	Head of dental department, orthodontist, senior lecturer, SDA president	Hospital, university, professional association	13 years	Outreach, advocacy, public awareness, professional leadership
CG01	Service-delivery participant	Senior dental clinician at tertiary teaching/referral hospital	Mogadishu Somalia-Turkiye Training and Research Hospital	5 years	High-volume clinical service, referral, data, workforce
CG02	Service-delivery participant	Dentist and private clinic manager	Private dental clinic/MCH-oriented service perspective	∼10 years	MCH linkage, affordability, HMIS, supplies
CG03	Service-delivery participant	Dentist and clinic owner	Private dental care facility	10 years	Service delivery, financing, referral, data gaps
CG04	Service-delivery participant	Hospital dental section respondent	Martini hospital dental section	Dental section established 2024	Public hospital service availability, workforce gaps
CG05	Service-delivery participant/social enterprise	Dentist at public-health-focused dental clinic	NDC Somalia/social enterprise model	>10 years	MCH integration, free care for children, EMR, infrastructure

### Data collection and preparation

Interview guides explored participants’ backgrounds and roles, current oral health services, policy and governance arrangements, financing mechanisms, workforce capacity, service delivery models, health information systems, commodities and infrastructure, key barriers and facilitators, and recommendations for integrating oral health into primary health care (PHC) and universal health coverage (UHC) reforms. The full interview guide is provided as Supplementary File S1. Interviews were conducted by a public health researcher with five years of professional experience in Somalia's health system. All 13 key-informant interviews were audio-recorded, transcribed verbatim, reviewed in full for accuracy, and de-identified using unique participant codes to protect confidentiality§.

### Analytical framework and coding

Data were analysed using a hybrid deductive-inductive thematic approach. Deductive coding domains were informed by the WHO health-system building blocks framework (16). Primary health care (PHC) principles, and the WHO global oral-health agenda. These frameworks provided an initial structure for examining governance, financing, workforce, service delivery, health information systems, medicines and technologies, and leadership factors relevant to oral health integration. Inductive coding was used to identify recurrent patterns and emerging issues within the interview data.

Key themes included policy-to-implementation gaps, weak regulatory oversight, reliance on out-of-pocket financing, workforce shortages and maldistribution, a predominance of hospital-based curative care, missed opportunities for integration within maternal and child health (MCH) and school-health programmes, limited visibility of oral health indicators within DHIS2, and feasible pathways for task-sharing and service integration at the primary care level.

Coding was managed using qualitative data-analysis software, NVivo version 14, to support systematic organization, retrieval, and comparison of coded data. Codes were developed iteratively and applied independently to a subset of transcripts by two members of the research team; coding was then compared, and any disagreements were resolved through discussion and consensus, with reference back to the original transcript and, where necessary, adjudication by a third member of the research team.

Codes were iteratively organized into broader themes and refined through constant comparison across transcripts. Particular attention was given to similarities and differences between the perspectives of system-level stakeholders and service-delivery providers. The resulting themes were triangulated with evidence from policy and technical documents and mapped to their implications for implementation, health-system strengthening, and the integration of oral health within PHC and universal health coverage (UHC) reforms in Somalia (17).

### Trustworthiness and reflexivity

Trustworthiness was enhanced through several strategies aligned with established qualitative research standards. Credibility was strengthened through triangulation across respondent groups and data sources, including policymakers, regulators, technical partners, academics, professional association leaders, and service-delivery stakeholders, as well as through comparison with findings from national and global policy documents. Credibility was further supported by independent double-coding of a subset of transcripts by two research team members, with disagreements resolved through discussion and consensus and, where necessary, adjudication by a third team member, and by continuing recruitment and data collection iteratively until no substantively new codes or themes emerged, at which point thematic saturation was judged to have been reached. Reporting was further guided by the COREQ checklist, provided in the Supplementary Materials. Dependability was supported by maintaining a transparent audit trail documenting coding decisions, subthemes, and theme development, and analytic refinements throughout the study. Confirmability was reinforced through the use of verbatim participant quotations to illustrate key themes and by clearly distinguishing descriptive findings from researchers' interpretive and policy-related inferences. Transferability was addressed through detailed contextual description of Somalia's health system, primary health care (PHC) reforms, and oral-health service landscape, enabling readers to assess the applicability of the findings to other fragile and resource-constrained settings.

With respect to reflexivity, the research team's positionality, including its familiarity with the policy environment and professional networks within the sector, was acknowledged as a potential influence on participant recruitment, rapport, and interpretation and was actively considered throughout data collection and analysis; interview questions were kept open-ended, and emerging interpretations were discussed within the research team to reduce the influence of individual assumptions on coding and theme development.

### Ethical considerations

Ethical approval for the study was obtained from the National Institute of Health (NIH) Ethics Review Board, Somalia, *(Reference No. NIH/SNREB/015/APRIL/2026),* in accordance with the ethical principles of the Declaration of Helsinki. All participants provided informed consent prior to participation after receiving information on the study objectives, procedures, the voluntary nature of participation, and their right to withdraw at any time without consequence. Confidentiality and participant privacy were protected through the anonymization of interview transcripts and the use of unique participant codes. All study data were stored securely and accessed only by authorized members of the research team.

## Results

The results are presented in four sections. First, we describe the policy and burden context that underpin the integration challenge. Second, we summarize the characteristics of the study participant. Third, we present the seven qualitative themes, comparing perspectives from policymaker and system-level stakeholders with those of service-delivery actors. Fourth, we map the findings to WHO health-system building blocks and propose a feasible pathway for integrating oral health services within the Somali primary health care (PHC) system.

### Policy and burden context

Somalia's policy framework (Table 1) provides a clear but yet underutilized opportunity for integration. The Health Sector Strategic Plan III (HSSP III) emphasizes integrated delivery of the Essential Package of Health Services (EPHS), universal health coverage (UHC), health security, equitable access, affordability, and quality care. The EPHS 2020 defines a package of preventive, promotive, curative, and rehabilitative services that can be progressively implemented across different levels of care. Similarly, the Somalia Investment Case prioritizes reforms that can accelerate EPHS delivery implementation within the existing resource constraints and promotes alignment among government institutions, development partners, private providers, and federal member states. Collectively, these policy instruments support the positioning of oral health as an integral component of PHC and UHC reforms rather than as a standalone specialist dental programme (9, 10, 18, 19).

This policy opportunity contrasts sharply with a substantial service-readiness gap. Available evidence indicates a high burden of untreated caries among both children and adults, alongside significant levels of severe periodontal disease and oral cancer. Despite this burden, key oral health services, including oral health screening, urgent oral care and pain management, and basic restorative treatment, were unavailable in public primary health care (PHC) facilities in 2021. the most recent year for which nationally comparable service-readiness data are available. No subsequent national oral health survey or facility-readiness assessment has since been published for Somalia.

This disconnect between population need and service capacity represents the central implementation challenge: although oral health is increasingly recognized as an important component of overall health and well-being, it remains inadequately operationalized within publicly financed PHC services in Somalia.

### Characteristics of the study participants

Table 2 presents the characteristics of the study participants. A total of 13 stakeholders were interviewed representing policy, regulatory, academic, professional, and service-delivery perspectives within Somalia's oral health system. Participants included a strategic technical advisor to the Ministry of Health, a senior regulator from the National Health Professional Council, dental academic leaders, and representatives of the Somali Dental Association. Frontline perspectives were provided by dentists and senior dental clinicians working in tertiary hospitals, private clinics, and public-health-oriented dental services. This diversity of participants provided perspectives from both strategic decision-making and frontline service delivery. The sample captured views from individuals involved in policy development, programme implementation, and health service utilization, enabling a comprehensive exploration of opportunities and barriers to integrating oral health services within primary health care (PHC) in Somalia. The inclusion of participants from different levels of the health system strengthened the breadth of insights generated through the thematic analysis.

### Overview of qualitative themes

Across the 13 transcripts, stakeholders consistently emphasized the importance of oral health and did not question its relevance within the health system. Rather, the dominant analytic narrative centred on implementation.

Participants described oral health as sufficiently visible to feature in policy discussions, yet insufficiently prioritized in practice through financing, workforce development, regulation, service delivery, monitoring systems, and supply-chain support. As a result, oral health remains only partially integrated into routine primary health care (PHC) services. Seven interrelated themes emerged from the analysis, highlighting the key opportunities and barriers shaping oral health integration in Somalia (Table 3).

**Table 3:** Final qualitative themes, subthemes and WHO health-system mapping.

Theme	Subthemes	WHO building block(s)	Analytic interpretation
1. Policy recognition without operationalization	EPHS/HSSP/UHC recognition; low prioritization; absence of budgeted implementation; weak public programme	Leadership/governance; financing; service delivery	Oral health is acknowledged in frameworks but not routinely delivered in PHC.
2. Fragmented governance, weak regulation, and limited coordination	Weak coordination; unclear standards; unlicensed practice; limited regulation of private clinics and training programmes	Leadership/governance; workforce; regulation; technologies	Integration requires a stronger stewardship function, formal standards, and enforcement.
3. Out-of-pocket financing and private-sector dependence	Private financing; absence of insurance; high OOP costs; delayed care; inequity for rural, poor, IDP, women and children	Health financing	Financing arrangements reproduce inequity and push patients toward late, more expensive care.
4. Workforce, education, and task-sharing constraints	Shortages and maldistribution; weak allied dental cadres; PHC workers not trained; hospital-centred curricula	Health workforce; service delivery	Dentist-only expansion is insufficient; PHC and community task-sharing is necessary but must be competency-based and supervised.
5. Curative, urban, hospital-centred service delivery	Advanced caries, periodontal disease, abscesses, trauma, and tooth loss; weak linkage to ANC, child health, school health, nutrition and NCD platforms	Service delivery; access; quality	Prevention, screening, triage, and referral must be embedded in routine PHC and MCH contacts.
6. HIS and evidence invisibility	Limited DHIS2 indicators; facility-level data not linked to national HMIS; weak workforce database; limited research infrastructure	Health information systems; governance	Without data, oral health remains invisible in planning, budgeting, and accountability.
7. Feasible phased integration pathways	PHC package; CHW/nurse/midwife training; referral protocols; school and MCH platforms; NCD alignment; commodity readiness	All building blocks	Stakeholders converged on a pragmatic, phased model rather than a parallel specialist programme.

### Theme 1. Policy recognition without operationalization

The first theme highlighted a persistent disconnect between policy recognition and practical implementation. Participants at the system-level reported that oral health is formally incorporated into national policy frameworks, including the Essential Package of Health Services (EPHS), Health Sector Strategic Plans (HSSPs), and broader non-communicable disease (NCD) strategies. However, this policy recognition has not been matched by adequate financing, operational guidance, service delivery standards, essential supplies, or routine integration within primary health care (PHC) services. Consequently, respondents characterized oral health as a policy priority in principle but not in practice, describing it as “policy on paper” with limited implementation at the service delivery level.

“Oral health is reflected in Somalia's national health policies, but its operationalization remains limited. A major challenge is that oral health has often been included in policy documents but not adequately funded or implemented.” [PM03]

“Yes oral health is part of EPHS but it is not implemented.” [PM01]

Service-delivery participant respondents described a similar implementation gap at the point of care. Oral health largely absent from routine service delivery within primary health care (PHC), including antenatal care (ANC), child health services, immunization clinics, nutrition programs, and community-based care. As a result, opportunities for prevention, early detection, and referral were frequently missed. Where oral health services were available, they were typically concentrated in hospitals, private clinics, or delivered through periodic outreach campaigns, rather than being integrated into a standardized and continuous PHC care pathway. This reinforced the perception that oral health remained a separate and higher-level service rather than a routine component of comprehensive primary care.

“Oral health services in maternal and child health care in Somalia are limited, with low integration, low awareness, and inadequate preventive services.” [CG02]

“There is currently no formal linkage or integration with PHC facilities.” [CG02]

### Theme 2. Fragmented governance, weak regulation, and limited coordination

The second theme concerned institutional fragmentation across the oral health system. Respondents described limited coordination among key stakeholders, including the Ministry of Health, professional councils, universities, private providers, hospitals, NGOs, and professional associations. This fragmentation was perceived as a major barrier to the effective integration and delivery of oral health services within primary health care (PHC). Respondents emphasized the need for stronger national leadership through standardized guidelines, regulatory enforcement mechanisms, multi-stakeholder coordination platforms, and clearer lines of accountability for implementation. Weak regulatory oversight was frequently associated with the proliferation of unqualified practitioners, unsafe tooth extractions, inadequate infection prevention and sterilization practices, and a consequent erosion of public confidence in oral health services. These challenges underscored the importance of strengthened governance and stewardship to ensure quality, safety, and consistency of care across sectors.

“Oral health coordination between the government, NGO partners and private sector is weak, there are no regular coordination activities and limited information sharing between stakeholders.” [PM03]

“Limited policy focus, workforce shortages, inadequate standards for PHC integration, and resource constraints.” [PM04]

“Coordination among the Ministry of Health, hospitals, PHC facilities, NGOs, and dental professionals is limited and needs strengthening to improve oral health integration.” [CG02]

Participants did not view regulation solely as a professional or administrative concern. Rather they linked regulatory oversight directly to patient safety, quality of care, and public confidence in oral health services. Respondents expressed particular concern about the widespread availability of low-cost services provided by informal or unqualified practitioners, especially in underserved areas where access to licensed dental care is limited. In such settings, procedures such as tooth extractions were often performed without adequate clinical training, proper equipment, or adherence to infection prevention and control standards. Participants perceived these practices as increasing the risk of complications, cross-infection, and poor treatment outcomes, while also undermining trust in the broader oral health system. Strengthening regulation and enforcement was therefore viewed not only as a mechanism for professional governance, but also as a critical strategy for safeguarding patient welfare and ensuring consistent standards of care.

### Theme 3. Out-of-pocket financing and private-sector dependence reproduce inequity

The third theme highlighted a financing structure characterized by heavy reliance on out-of-pocket expenditure and private-sector provision. Policymakers described chronic underinvestment in oral health, noting the limited allocation of domestic health resources, the absence or limited coverage of dental services within health insurance schemes, and the relatively low priority accorded to oral health within donor-funded health programs. As a result, oral health services were often perceived as discretionary rather than essential, limiting their integration into broader health financing mechanisms.

Service-delivery participants described the direct consequences of these financing barriers at the household level. The cost of consultations, treatment, transportation, and medications frequently discouraged timely care-seeking, particularly among low-income families. Consequently, many individuals delayed seeking treatment until symptoms became severe, often relying on self-medication, traditional remedies, or informal providers as more affordable alternatives. Participants reported that conditions that could have been prevented or managed through early intervention commonly progressed to pain, infection, tooth loss, functional impairment, and the need for more complex and costly treatment. In our interpretation, these qualitative accounts suggest that financial barriers not only reduce access to oral healthcare but also contribute to the worsening severity of oral diseases and the accumulation of unmet treatment needs within the population.

“Oral health financing is largely out of pocket except limited hospitals that provide subsidized oral health services in public hospitals.” [PM03]

“Private only.” [PM01]

“Public health facilities rarely receive state funding for dental equipment or staff, meaning that a family's financial situation completely dictates their access to care.” [CG05]

The equity implications of the prevailing financing model emerged as a major concern across respondent groups. Participants emphasized that financial barriers disproportionately affect populations that already experience social, geographic, and economic disadvantage, including low-income households, rural and nomadic communities, internally displaced persons, women, and children. These groups often face multiple constraints to accessing oral healthcare, such as limited service availability, transportation challenges, reduced financial capacity, and competing household priorities. Consequently, oral diseases frequently remain untreated, contributing to avoidable pain, disability, and wider health and social inequities.

Respondents argued that achieving equitable access to oral healthcare would require the adoption of more inclusive and sustainable financing mechanisms. Several strategies were proposed, including the incorporation of essential oral health services into Universal Health Coverage (UHC) benefit packages, the expansion of social health insurance schemes to cover preventive and curative dental care, and the introduction of targeted voucher programs for vulnerable populations. Participants also recommended subsidized emergency oral health services to reduce financial hardship associated with acute dental conditions. At the system level, respondents highlighted the need for increased domestic resource mobilization and dedicated budget allocations for oral health. In addition, some participants advocated for the use of health-promoting fiscal policies, such as taxes on sugar-sweetened beverages and tobacco products, with revenues earmarked to support oral health promotion, disease prevention, and service delivery. Collectively, these approaches were viewed as important mechanisms for reducing financial barriers, strengthening equity, and advancing the integration of oral health within broader UHC reforms.

### Theme 4. Workforce, education, and task-sharing constraints

The fourth theme focused workforce capacity, distribution, competency, and regulation as critical determinants of oral health service delivery. Respondents consistently reported shortages of dentists and specialists, particularly outside major urban centers, alongside a limited number of allied oral health personnel capable of supporting preventive and primary-level services. Workforce maldistribution was identified as a significant barrier to access, with rural, remote, and underserved communities facing persistent shortages of qualified providers. As a result, access to routine oral healthcare was often constrained by distance, travel costs, and long waiting times, further exacerbating existing inequalities.

“PHC staff are not trained in the BPOC services developed yet.” [PM03]

“Training non-dental PHC workers is essential to expand access to preventive oral health services, early detection, and timely referral.” [PM04]

“The current dental curriculum does not adequately prepare graduates. It is typically very hospital-centered and focused on specialized clinical procedures.” [AC02]

Service-delivery respondents further emphasized that workforce challenges extend beyond shortages of dentists alone. Participants reported insufficient numbers of dental therapists, dental hygienists, dental nurses, dental technicians, and specialist practitioners, creating significant constraints across all levels of service delivery. In many facilities, particularly those with limited staffing, dentists were required to assume responsibilities that would normally be undertaken by auxiliary personnel, including preventive services, patient education, administrative tasks, and basic clinical support functions. This concentration of responsibilities was perceived as reducing efficiency, increasing provider workload, and contributing to service bottlenecks in already overstretched clinics. Respondents noted that these workforce limitations often resulted in longer waiting times, reduced service capacity, and diminished opportunities to expand preventive and community-based oral healthcare.

Within this context, task-sharing was widely viewed as a practical and necessary strategy for strengthening oral health services and supporting their integration within primary health care (PHC). Participants highlighted the potential role of allied oral health personnel and other PHC workers in delivering preventive interventions, oral health education, screening, basic management, and referral services. However, respondents stressed that task-sharing should be implemented within a robust governance framework to ensure quality and patient safety. Key requirements included clearly defined scopes of practice and competency standards, appropriate training and certification, ongoing supervision and mentorship, standardized referral pathways, adherence to infection prevention and control protocols, and strong regulatory oversight. Participants emphasized that, when adequately supported, task-sharing could improve service accessibility, efficiency, and workforce sustainability while maintaining acceptable standards of care across diverse settings.

### Theme 5. Service delivery is curative, urban, hospital-centred, and weakly linked to MCH/PHC platforms

The fifth theme highlighted a predominantly treatment-oriented model of oral healthcare, characterized by high volumes of advanced dental caries, periodontal disease, abscesses, impacted teeth, trauma, and premature tooth loss. Clinical respondents reported that patients commonly presented only after significant disease progression, often requiring complex and invasive treatment. Participants attributed these late presentations to low oral health awareness, financial barriers to care, limited availability of preventive services, and weak oral health screening and triage within primary health care (PHC) settings. As a result, the system was perceived as largely reactive rather than preventive, with missed opportunities for early detection, health promotion, and timely intervention contributing to a substantial burden of avoidable oral disease.

“The vast majority of patients present with deep, untreated cavities that have already progressed to acute pulpitis or periapical abscesses, frequently requiring root canal therapy or extraction.” [CG01]

“Most patients come for treatment after problems develop, so curative care is more common than preventive care.” [CG02]

“No oral health services provided at PHC level except few irregular oral health campaigns in schools and other community settings.” [PM03]

Service-delivery participant respondents highlighted substantial missed opportunities to integrate oral health into existing maternal and child health platforms. Services such as antenatal care (ANC), immunization programs, nutrition services, child health clinics, school health programs, adolescent health services, and non-communicable disease (NCD) clinics were repeatedly identified as strategic entry points for oral health promotion and early intervention. Participants suggested that these platforms could incorporate counselling on oral hygiene practices, dietary and sugar-risk reduction, promotion of fluoride toothpaste use, basic oral health screening, and referral pathways with relatively modest additional investment. Respondents viewed such integration as a practical and cost-effective approach to expanding preventive oral healthcare, improving early detection of oral diseases, and strengthening the role of primary health care (PHC) in oral health service delivery.

“You will not find them in Antenatal Care checkups, pediatric wellness visits, nutritional monitoring programs, school health initiatives, or immunization clinics.” [CG05]

### Theme 6. Oral health remains invisible in health information systems and evidence generation

The sixth theme concerned information systems, evidence generation, and planning. Respondents described an information gap in oral health data collection and use, noting that oral health indicators are inadequately represented within DHIS2, routine facility reporting systems, workforce databases, private-sector reporting mechanisms, and national surveillance activities. Although some facilities maintain local electronic records or spreadsheets, these data are often fragmented and not integrated into national health information systems, limiting their usefulness for planning, resource allocation, and performance monitoring. Participants emphasized that the absence of reliable and comprehensive oral health data weakens evidence-based decision-making, obscures the true burden of disease, and constrains efforts to advocate for resources, monitor service delivery, and guide the integration of oral health within primary health care (PHC).

“Currently only dental consultations as one indicator is reported through the DHIS2.” [PM03]

“No. Oral health data is not routinely collected in Somalia's DHIS2 or any other national health information system.” [PM05]

“We collect and store oral health data in our own electronic system for patient management. However, we are not connected to the government HMIS.” [CG02]

Academic respondents linked these information gap to broader weakness in research capacity evidence generation. Participants reported limited collaboration between universities and service-delivery sites, a lack of population-level oral-health surveys, insufficient access to public hospital data and constrained resources for oral health research. As a result, policymakers and planners often lack the evidence needed to assess disease burden, evaluate service performance, and inform priority setting. Respondents described this as a self-reinforcing cycle in which oral health remains inadequately measured and therefore largely invisible within health planning, budgeting, and policy processes. In turn, its low visibility reduces investment in surveillance, research, and health information systems, perpetuating the scarcity of data needed to support evidence-based decision-making and the integration of oral health within primary health care (PHC).

### Theme 7. Integration is feasible if built onto existing PHC, MCH, school, CHW, and NCD platform

Despite substantial barriers, participants converged around a pragmatic integration and feasible model for oral health integration. Rather than advocating for the immediate establishment of separate and resource-intensive dental infrastructure in every district, respondents favored a phased approach that builds on existing health system platforms. Oral health services were viewed as most effectively delivered through established primary health care (PHC), maternal and child health (MCH), school health, community health, nutrition, non-communicable disease (NCD), and referral services. Participants suggested that these platforms could progressively incorporate preventive interventions, oral health education, basic screening, risk assessment, early referral, and selected essential oral health services. This approach was perceived as a cost-effective and scalable strategy that leverages existing workforce, infrastructure, and service delivery mechanisms while expanding access to oral healthcare and strengthening integration within the broader health system (Figure 1).

**Figure 1:**
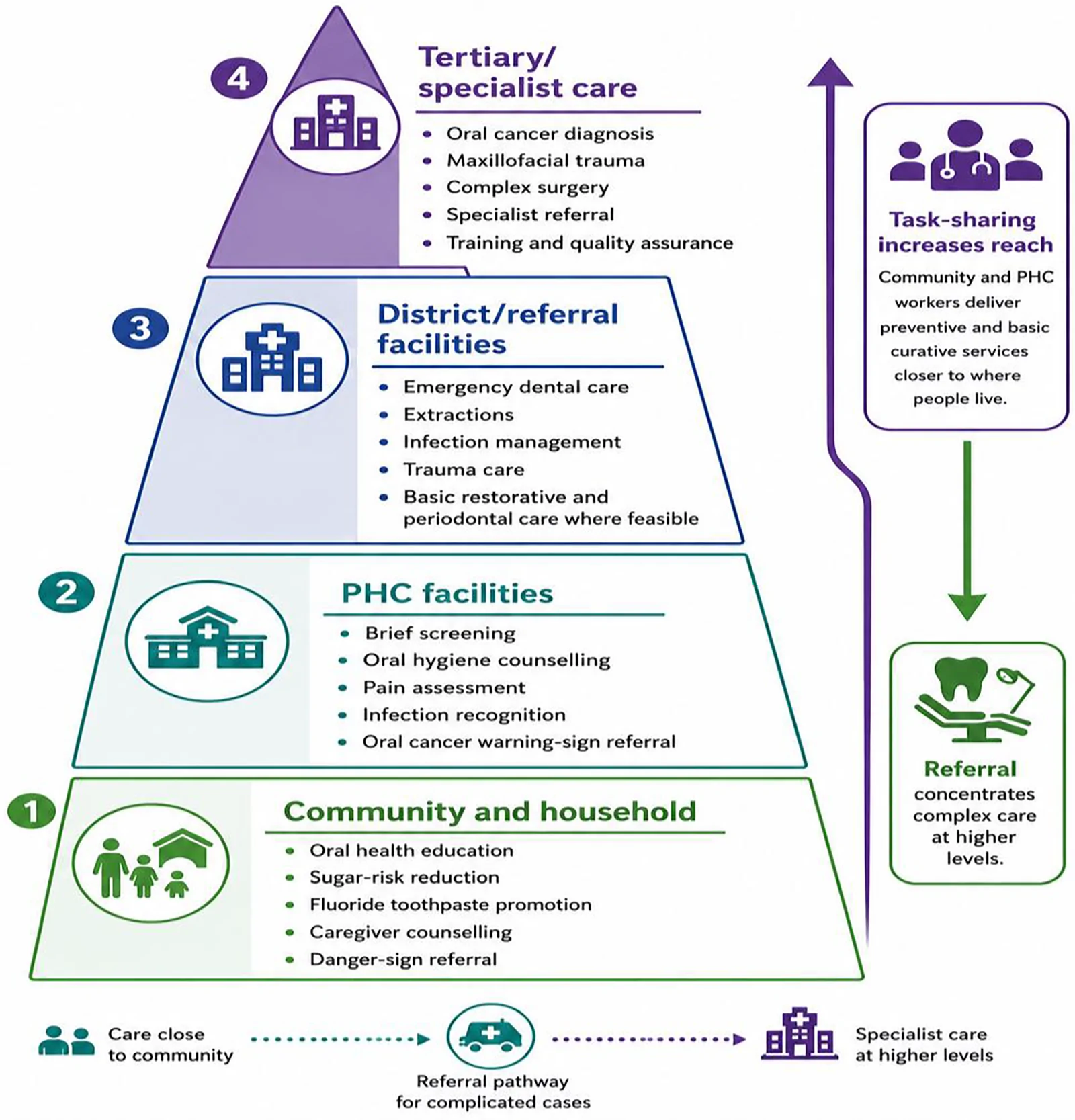
Tiered oral health service-delivery model for Somalia.

“It's feasible, for example, to provide basic oral health services in the existing PHC services. This requires government commitment, and provision of necessary training and supplies to the PHC facilities/health workers.” [PM02]

“Oral health can be integrated into ANC, child health, nutrition, and immunization services by adding basic screening, providing oral health education, offering preventive care, and strengthening referral systems to dental clinics.” [CG02]

“School health programs and community health workers provide major opportunities.” [CG03]

Respondents across stakeholder groups proposed a broadly consistent and pragmatic package for integrating oral health into the health system (illustrative supporting quotations are provided in Table 4). Key components included the development of a national oral health policy or implementation roadmap, establishment of a minimum package of basic oral healthcare within primary health care (PHC), and targeted training for community health workers, nurses, midwives, PHC physicians, teachers, and nutrition personnel. Participants also emphasized the importance of standardized referral protocols, availability of essential commodities and infection-prevention supplies, public awareness and health-promotion campaigns, inclusion of oral health indicators within DHIS2 and routine health information systems, and strengthened regulation of oral health providers.

**Table 4:** Supplementary (Supplementary Table S1) illustrative quotations by theme.

Theme/domain	Participant code	Representative quotation
Policy recognition without operationalization	PM03	Oral health is reflected in Somalia's national health policies, but its operationalization remains limited. A major challenge is that oral health has often been included in policy documents but not adequately funded or implemented.
Policy recognition without operationalization	PM01	Yes oral health is part of EPHS but it is not implemented.
Weak PHC/MCH linkage	CG02	Oral health services in maternal and child health care in Somalia are limited, with low integration, low awareness, and inadequate preventive services.
Governance and coordination	PM03	Oral health coordination between the government, NGO partners and private sector is weak, there are no regular coordination activities and limited information sharing between stakeholders.
Financing and equity	PM03	Oral health financing is largely out of pocket except limited hospitals that provide subsidized oral health services in public hospitals.
Financing and equity	CG05	Public health facilities rarely receive state funding for dental equipment or staff, meaning that a family's financial situation completely dictates their access to care.
Workforce and task-sharing	PM04	Training non-dental PHC workers is essential to expand access to preventive oral health services, early detection, and timely referral.
Curriculum and education	AC02	The current dental curriculum does not adequately prepare graduates. It is typically very hospital-centered and focused on specialized clinical procedures.
Curative service model	CG01	The vast majority of patients present with deep, untreated cavities that have already progressed to acute pulpitis or periapical abscesses, frequently requiring root canal therapy or extraction.
Missed MCH/PHC opportunities	CG05	You will not find them in Antenatal Care checkups, pediatric wellness visits, nutritional monitoring programs, school health initiatives, or immunization clinics.
HIS invisibility	PM03	Currently only dental consultations as one indicator is reported through the DHIS2.
Feasible integration	CG02	Oral health can be integrated into ANC, child health, nutrition, and immunization services by adding basic screening, providing oral health education, offering preventive care, and strengthening referral systems to dental clinics.

### Policymaker vs. service-delivery participant comparison

Table 5 highlights a strong convergence between policymaker/system-level and service-delivery perspectives, with both groups identifying similar underlying challenges while emphasizing different levels of the health system. Policymakers focused on upstream determinants, including the weak operationalization of oral health policies, inadequate financing, workforce planning gaps, fragmented data systems, and deficiencies in regulation and quality assurance. Service-delivery participant, by contrast, described the downstream consequences of these shortcomings, including the absence of oral health within routine PHC and MCH pathways, unaffordable out-of-pocket costs, high patient loads, shortages of skilled personnel and supplies, emergency-driven attendance, weak referral mechanisms, and concerns about unsafe informal practice. As shown in Table 5, these perspectives are complementary rather than contradictory: policy-level failures in financing, workforce development, service organization, information systems, and regulation are reflected in delayed care-seeking, advanced disease at presentation, pressure on tertiary services, and missed opportunities for prevention. Together, the findings suggest that successful oral health integration will require simultaneous action at both system and service-delivery levels, linking policy reform with frontline implementation.

**Table 5:** Policymaker vs. service-delivery participant perspectives.

Domain	Policymaker/system-level emphasis	Service-delivery emphasis	Interpretive comparison
Policy positioning	EPHS/HSSP/UHC inclusion but weak operationalization; need for policy, roadmap, standards and regulation.	No routine PHC/MCH linkage; oral health absent from patient pathways in ANC, immunization, nutrition and child health.	Shared view that oral health is recognized but not embedded in routine care.
Financing	Public funding gaps, UHC benefit packages, social insurance, donor financing, and domestic resource mobilization.	Unaffordable care, OOP payments, delayed attendance and financial distress among families.	Policy-level financing failure translates into delayed and more severe clinical presentations.
Workforce	Competency standards, accreditation, curricula, BPOC, and task-sharing for CHWs, nurses and midwives.	High patient load, lack of allied cadres, practical skill gaps, and shortage of specialists.	Integration requires both national standards and service-level staffing capacity.
Service delivery	Phased PHC package, prevention, triage, referral, and use of existing platforms.	Advanced disease, emergency-driven attendance, and weak referral from PHC to dental care.	Prevention and triage would reduce tertiary bottlenecks.
HIS/evidence	DHIS2 indicators, workforce data, private-sector reporting, and national surveys.	Facility records or EMRs exist but are disconnected from government HMIS.	Routine indicators are needed to convert local evidence into national planning.
Regulation and quality	Licensing, accreditation, enforcement, and standards for private providers and training institutions.	Unsafe informal practice and quality concerns lead to complications and distrust.	Regulation is a patient-safety prerequisite, not only a professional issue.

Table 6 maps the study findings against the WHO health-system building blocks and highlights their implications for oral health integration. Across all building blocks, respondents described systemic weaknesses that collectively constrain the delivery of accessible, equitable, and preventive oral health services. Respondents identified weak leadership and governance, inadequate financing, workforce shortages, fragmented service delivery, insufficient infrastructure and supplies, and limited health information systems as interconnected barriers to effective oral health care. Oral health was perceived as policy-recognized but poorly implemented, heavily reliant on out-of-pocket payments, concentrated in urban hospital settings, and constrained by shortages of trained personnel, equipment, and routine data. These weaknesses were further compounded by poor coordination, weak regulation, unclear referral pathways, and limited integration of oral health into PHC, MCH, school health, nutrition, and NCD platforms. The findings suggest that successful integration will require a comprehensive health-systems approach, including stronger governance and regulation, sustainable financing within EPHS and UHC frameworks, competency-based workforce development, expanded preventive and referral services at PHC level, improved infrastructure and supply chains, and the incorporation of oral health indicators into national health information systems.

**Table 6:** WHO health-system building-block mapping and implementation implications.

WHO building block	Findings from qualitative data	Integration implication
Leadership and governance	Policy inclusion without implementation; weak coordination; weak regulation and standards; unqualified practice; limited leadership prioritization.	Develop national oral health policy/roadmap; define PHC standards; establish MoH-NHPC-SDA-university coordination; enforce licensing and accreditation.
Health financing	OOP/private financing; no insurance; limited domestic and donor funding; affordability barriers for poor, rural, women, children and IDP populations.	Include basic preventive and emergency oral care in EPHS/UHC financing; explore vouchers, social insurance, subsidies, and taxation of risk-related products.
Health workforce	Shortages, maldistribution, limited allied cadres; PHC workers not trained; hospital-centred dental education; weak accreditation and CPD.	Competency-based training for CHWs, nurses, midwives and PHC doctors; strengthen dental hygienist/therapist cadres; harmonize curricula and CPD.
Service delivery	Curative, urban, hospital-centred care; limited prevention; weak ANC, child health, nutrition, school health and NCD integration; unclear referral pathways.	Introduce education, screening, triage, referral and selected emergency care through PHC/MCH/school/NCD/community platforms.
Medical products, technologies and infrastructure	Limited dental rooms, chairs, x-ray, sterilization, water, electricity, consumables, and maintenance; dependence on imports and shipping delays.	Define staged commodity package; include oral-health supplies in EPHS supply planning; strengthen IPC, maintenance and low-resource/mobile models.
Health information systems	Oral health not systematically captured in DHIS2; private providers do not routinely report; local data are disconnected; limited research infrastructure.	Pilot minimal DHIS2 indicators; map workforce and providers; require private-sector reporting; conduct national oral-health survey and facility readiness assessments.

Overall, Table 6 demonstrates that oral health integration is a system-wide challenge requiring coordinated action across all WHO health-system building blocks. The findings indicate that effective integration will depend not only on expanding clinical services, but also on strengthening governance, financing, workforce capacity, infrastructure, supply systems, and health information systems to support sustainable and equitable oral health care within PHC and UHC frameworks.

## Discussion

This integrated analysis shows that oral health integration in Somalia is best understood as fundamentally a health-systems implementation challenge (Figure 2) rather than a problem of unmet need or complete policy neglect. While oral health is increasingly recognized within national policies and strategic frameworks, substantial gaps remain between policy intent and implementation across governance, financing, workforce, service delivery, commodities, regulation, and information systems. (Themes 1–6). The qualitative findings complement the policy analysis by illustrating how these systemic weaknesses are experienced at the frontline manifesting as unaffordable care, delayed care-seeking, emergency- and pain driven attendance, overburdened hospital services, weak referral mechanisms, low public awareness, shortages of essential supplies, and absent of routine oral health screening and prevention within maternal and child health (MCH) and primary health care (PHC) services.

**Figure 2:**
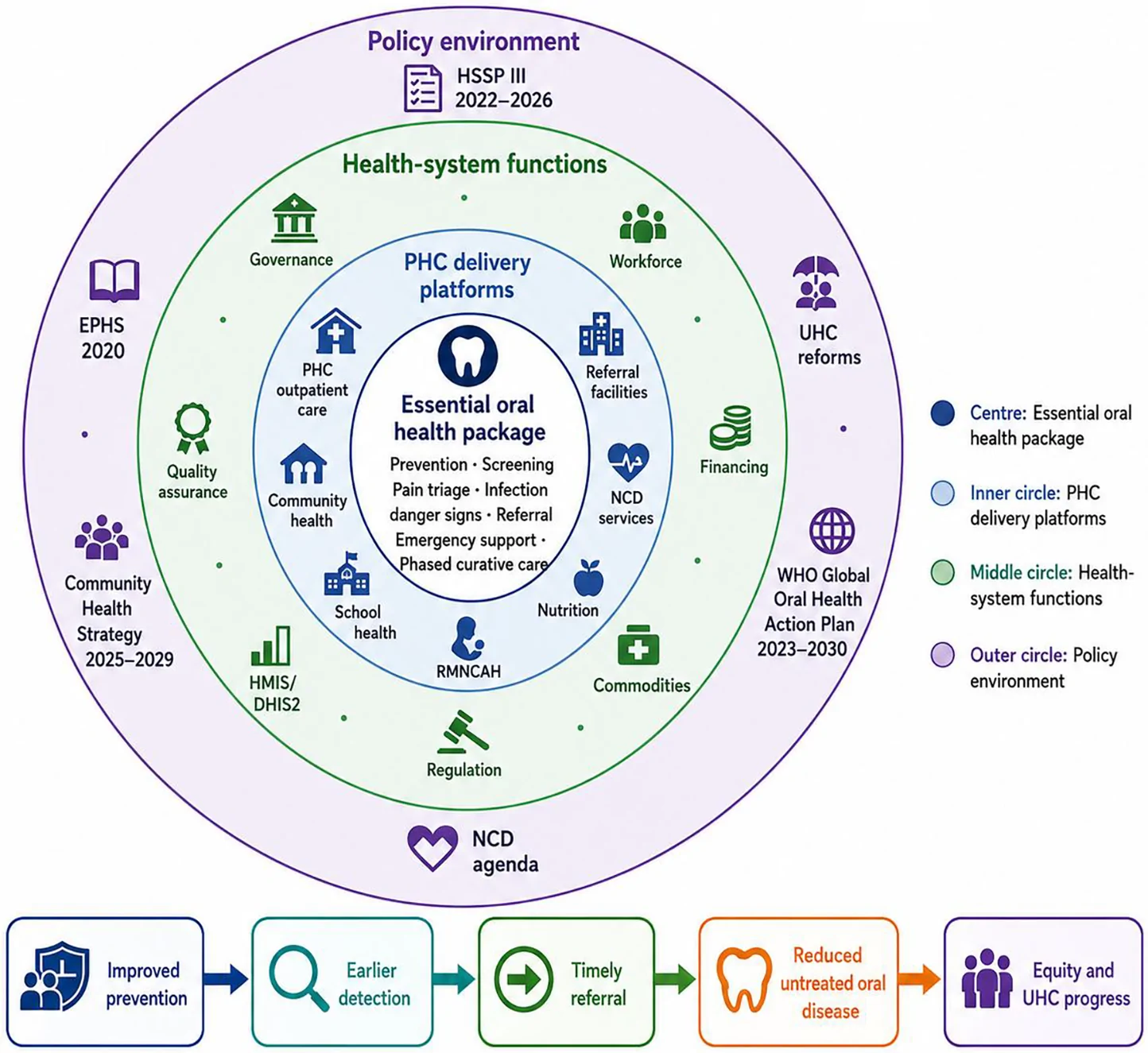
Multilevel conceptual framework for integrating oral health into Somalia's PHC and UHC agenda.

Beyond documenting these barriers individually, the findings point to important interactions among the WHO health-system building blocks that help explain why oral health integration has stalled despite policy recognition. Weak governance and coordination (Theme 2) limit the Ministry of Health's capacity to translate policy commitments (Theme 1) into binding financing decisions (Theme 3), so that oral health competes poorly for scarce domestic and donor resources against services with clearer lines of accountability. This financing gap, in turn, constrains pre-service and in-service investment in the oral health workforce (Theme 4), reinforcing a small, urban, dentist-centred cadre that can sustain only a curative, hospital-based model of care (Theme 5). The same governance weaknesses that limit regulation of private providers also limit the institutional mandate to require standardized reporting, helping to explain why oral health indicators remain largely absent from DHIS2 (Theme 6) despite longstanding policy recognition. The seven themes are best understood not as independent barriers but as a mutually reinforcing cycle, in which weak stewardship perpetuates under-financing, under-financing perpetuates workforce and service-delivery gaps, and the resulting absence of routine data further weakens the evidence base needed to advocate for stronger stewardship and financing. Political and institutional factors reinforce this cycle: oral health lacks a dedicated budget line within the Ministry of Health, and a constituency comparable to that of maternal, child, or communicable-disease programmes, so it is repeatedly deprioritized during resource allocation even where it is formally included in policy documents. Interrupting this cycle at a single point, such as expanding the workforce alone, is therefore unlikely to be sufficient; the phased pathway proposed under Theme 7 was designed to act on governance, financing, workforce, and information systems concurrently rather than to service expansion in isolation.

A second key interpretation, drawn from the convergence of respondent views (Theme 7), is that oral health integration in Somalia is unlikely to succeed through a traditional dentist-centred, clinic-based expansion model alone. Such an approach would be costly, concentrated in urban areas, constrained by workforce shortages, and poorly aligned with the principles of PHC. Instead, respondents from both policy and service-delivery levels advocated a phased pathway that begins with policy development, governance strengthening, and system readiness, followed by pilot integration within PHC and community platforms, establishment of effective referral and regulatory mechanisms, and gradual expansion of financing and service coverage. This phased approach is consistent with Somalia's Essential Package of Health Services (EPHS) framework and broader health-sector reform agenda, which emphasize incremental implementation based on available resources and system capacity (20, 21).

A third important finding is that task-sharing (Theme 4) is a necessary but insufficient strategy for expanding access to oral health services. Community health workers (CHWs) and Female Community Health Workers(Marwo Caafimaad), nurses, midwives, nutrition workers, teachers, and NCD clinicians could play a critical role in delivering prevention, health education, risk assessment, screening, counselling, and referral services (11). However, the effectiveness and safety of task-sharing depend on supporting system investments, including national competency frameworks, standardized training materials, supportive supervision, referral protocols, infection prevention and control measures, and regulatory oversight. Without these enabling structures, task-sharing risks increasing demand for care without ensuring access to safe, affordable and quality treatment services (13).

### Interpretation in relation to global and national policy

The findings are consistent with WHO's global oral-health agenda, which positions oral health as an integral component of universal health coverage (UHC), primary health care (PHC), non-communicable disease (NCD) prevention, surveillance, and broader health-system strengthening efforts. They also reflect growing international concern that oral diseases remain among the most prevalent yet neglected health conditions worldwide, despite being largely preventable, socially patterned, and associated with substantial health and economic consequences (3, 22). In Somalia these global challenges are further intensified by a fragile health system, ongoing reconstruction of public-sector infrastructure, constrained government and donor resources, and a heavy reliance on private providers for dental service delivery. Consequently, improving oral health requires not only service expansion but also strategic integration within existing health-system reforms and service platforms.

These findings resonate with experiences reported elsewhere in fragile and resource-constrained health systems that have attempted to integrate oral health into primary care. In Kenya, recent health-sector reforms, including the 2023 Primary Health Care Act and the Social Health Authority's benefit package, have created policy openings for oral health integration, yet implementation remains constrained by discordant governance arrangements, inadequate financing, and a maldistributed workforce that closely mirror the barriers identified in the present study (23). Similarly, a policy landscape analysis in Ethiopia found that despite national commitment to health-sector development plans, oral health remains poorly integrated into the broader health system and is largely excluded from insurance coverage, leaving cost and access as the principal barriers faced by patients (24). In Rwanda, oral health practitioners have reported managerial and systemic barriers, including the neglect of dental services during recruitment and procurement, that similarly reflect weak organizational planning rather than an absence of policy commitment (25). Taken together, these examples suggest that the implementation gap identified in Somalia is not unique but reflects a broader pattern across fragile and transitional health systems, in which oral health policy commitments outpace the governance and financing structures needed to operationalize them.

Brazil offers a contrasting example of what sustained political commitment and structural integration can achieve. Since 2004, oral health teams have been embedded directly within Brazil's Family Health Strategy, the organizing platform for primary care under its universal health system, with population coverage expanding substantially over the subsequent decade under the “Smiling Brazil” national oral health policy (26). This experience suggests that the phased, PHC-embedded pathway proposed in the present study is achievable at scale, but only when accompanied by durable financing commitments and a primary care workforce structure designed from the outset to include oral health personnel conditions that remain largely absent in Somalia. Nepal offers a more cautionary parallel: despite a dedicated National Oral Health Policy and more than two decades of experience delivering a Basic Package of Oral Care through mid-level providers, routine, system-wide delivery has not been achieved, in part because of the absence of standardized competency frameworks and regulatory recognition for non-dental providers delivering oral care (27). This mirrors the present study's finding that policy recognition alone, without accompanying workforce standards and regulatory mechanisms, is insufficient to sustain integration. Considered together, the comparative evidence suggests that the barriers identified in Somalia are largely generalizable to fragile and resource-constrained health systems attempting oral health-PHC integration, while the scale of Somalia's post-conflict, aid-dependent context is likely to make the pathway to integration slower and more incremental than in middle-income settings such as Brazil.

Importantly, the current policy environment presents a significant opportunity for integration. National frameworks, including HSSP III, the EPHS 2020, the Somalia Health Sector Investment Case, and ongoing community health reforms, provide potential entry points for embedding oral health within existing programmes rather than establishing a separate vertical system. Respondents identified several practical integration pathways. Within reproductive, maternal, newborn, child and adolescent health (RMNCAH), oral health could be incorporated through maternal counselling, promotion of healthy infant feeding practices, and prevention of early childhood caries. Within nutrition programmes, oral health messages could support sugar-risk reduction, while referral mechanisms could address oral conditions that contribute to feeding difficulties. Integration with NCD services could focus on shared risk factors, tobacco cessation, diabetes–periodontal health linkages, and early recognition of oral cancer warning signs. Similarly, school health programmes could provide platforms for oral hygiene education, preventive interventions, and routine screening (10, 17, 28).. These opportunities suggest that oral health can be mainstreamed within existing PHC structures, thereby increasing reach, efficiency, and sustainability while avoiding competition for scarce resources. Overall, the findings indicate that oral health integration in Somalia is most likely to succeed when embedded within broader health-system strengthening and multisectoral service-delivery initiatives.

In addition, given Somalia's heavy reliance on private-sector dental provision, governance of this sector will need to extend beyond regulation and accreditation alone. Effective licensing, accreditation, reporting, and referral requirements remain necessary to improve quality, patient safety, infection prevention, and continuity of care, and to reduce risks associated with unregulated practice. However, international experience with private-sector engagement and provider payment reform indicates that regulation alone is rarely sufficient to align private-sector behaviour with public health goals, particularly where regulatory and monitoring capacity is limited (29, 30). Strategic purchasing arrangements including selective contracting of qualified private providers, output- or performance-based payment for defined preventive and essential curative services, and public-private partnership models in which government funds or vouchers are used to purchase oral health services from accredited private providers could allow the Ministry of Health to leverage existing private-sector capacity for population-level coverage while retaining stewardship over quality and equity. Such mechanisms would need to be phased in alongside the governance and information-system strengthening described above, since effective contracting and purchasing depend on the same institutional capacities, a clear regulatory mandate, reliable provider data, and monitoring infrastructure that this study found to be currently underdeveloped.

Broader implications for health system strengthening. Although this study focused on oral health, the barriers identified here, formal policy recognition unmatched by financing, workforce investment, regulation, or routine data collection, are not unique to oral healthcare. Similar patterns have been described in other historically neglected areas of Somalia's health system, including mental health, eye and vision care, rehabilitation and disability services, and palliative care, which compete for the same constrained fiscal space and are similarly absent from routine PHC pathways and health information systems. The phased, PHC-embedded model proposed here, strengthening governance and a defined minimum service package before expanding curative capacity, may therefore offer a transferable template for integrating other neglected services into Somalia's PHC platform. A common thread across these neglected areas is the need for multi-disciplinary teams at the primary-care level, combining community health workers, nurses, midwives, teachers, and NCD clinicians, who can jointly deliver prevention and health-promotion messaging (for example, oral health, mental health, vision, and NCD risk-factor screening) through a single community contact, rather than requiring separate cadre-specific outreach for each condition. Such multi-disciplinary, task-shared models are consistent with WHO's broader PHC revitalization agenda and could improve the efficiency and reach of prevention and promotion services across several currently neglected domains simultaneously. The interview guide and COREQ checklist underpinning the qualitative findings that inform this discussion are provided in the Supplementary Materials.

### Policy and programme implications

The findings suggest that integrating oral health into Somalia's health system is both necessary and feasible but will require a phased and system-oriented approach. To enhance the practical value of these recommendations for policymakers, we organize them below into short-term, medium-term, and long-term priorities within a phased implementation roadmap, recognizing that each phase builds on the governance and information-system foundations established in the phase before it.

Short-term priorities (approximately 0–2 years) should focus on establishing the governance and service foundations needed for integration. At the policy level, stronger leadership and governance are needed to position oral health within national PHC, EPHS, NCD, and UHC agendas; strengthening the oral health unit within the Ministry of Health would provide strategic direction, facilitate stakeholder engagement, and guide implementation through a national integration roadmap. From a programme perspective, an immediate priority is to define a minimum package of preventive and promotive oral health interventions that can be delivered at primary care and community levels through EPHS implementation, prioritizing health promotion, risk-factor reduction, early detection, triage, and referral. In parallel, oral health indicators should be incorporated into national health information systems, beginning with a limited and feasible DHIS2 indicator set covering screening, counselling, referrals, school-based activities, and service readiness, to generate evidence for decision-making and facilitate gradual scale-up. Initial workforce efforts should prioritize competency-based training of community health workers and other PHC providers to deliver this minimum package while maintaining clear professional boundaries that preserve the clinical responsibilities of dental practitioners. Establishing baseline regulatory functions for private and informal dental services, including licensing, accreditation, reporting, and referral requirements, should also begin in this phase to improve quality, patient safety, and continuity of care.

Medium-term priorities (approximately 2–5 years) should build on this foundation through workforce and financing reforms. Sustained investment in pre-service education, practical training, community-based learning, and public health dentistry capacity will be needed to expand and diversify the oral health workforce beyond the initial cohort of trained community health workers. Financing reforms should mobilize domestic and donor resources for cost-effective preventive interventions, workforce development, essential supplies, referral systems, and emergency care for vulnerable populations. As governance and information-system capacity mature, strategic purchasing arrangements, including selective contracting of qualified private providers, performance-based payment for defined services, and public-private partnership models could be introduced to leverage existing private-sector capacity for population-level coverage while retaining stewardship over quality and equity.

Long-term priorities (5 years and beyond) should focus on consolidating and sustaining these gains. Expansion to curative services should be linked to demonstrated local readiness, workforce availability, and quality assurance mechanisms established during the earlier phases. As broader health financing reforms advance, oral health services should be progressively incorporated into UHC benefit packages to improve financial protection and equitable access to care.

Overall, this phased roadmap moving from foundational governance, minimum service packages, and data systems in the short term, through workforce and financing reform in the medium term, to full curative-service expansion and UHC integration in the long term provides a practical sequence for policymakers seeking to strengthen oral health outcomes in Somalia (Figure 3).

**Figure 3:**
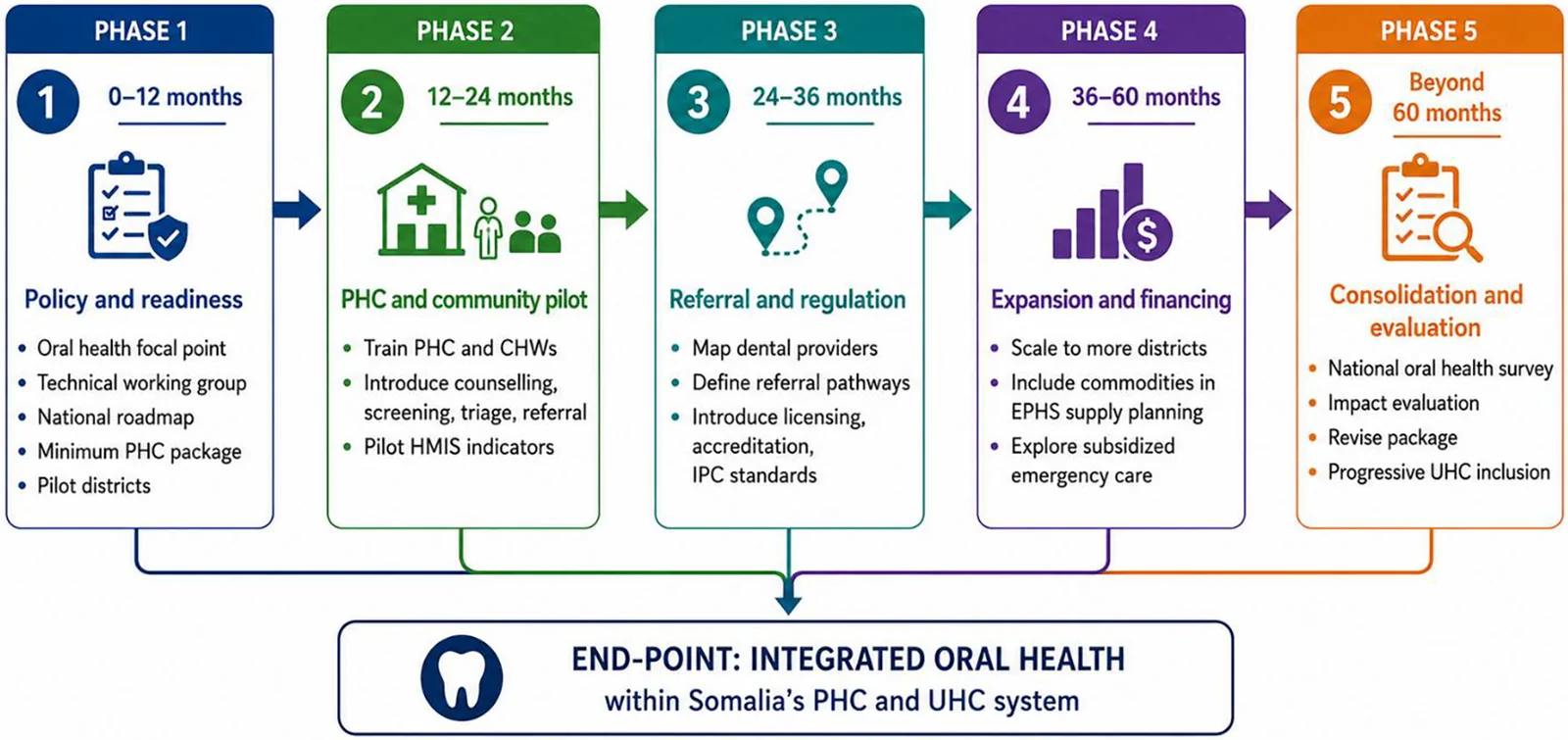
Phased implementation pathway for oral health integration.

### Strengths and limitations

This study has several important strengths. First, it combines national policy and document analysis with qualitative evidence from a diverse group of stakeholders, including policymakers, technical partners, regulators, academics, professional association leaders, and service-delivery respondents. This approach enabled triangulation of findings across multiple perspectives and sources. Second, the analysis was structured using the WHO health-system building blocks framework, providing a comprehensive and policy relevant assessment of opportunities and barriers to oral health integration. Third, the study moves beyond problem identification by translating findings into practical policy and programme recommendations that are tailored to Somalia's fragile health-system context. Finally, the inclusion of verbatim quotations enhances the credibility and transparency of the analysis by allowing readers to assess the relationship between the underlying data and the study's interpretations.

The study also has several limitations. The study also has several limitations. The qualitative dataset consisted of 13 transcripts and therefore may not fully represent the perspectives of all federal member states or key population groups, including, rural and nomadic communities, internally displaced persons (IDPs), patients, primary health care workers, or community health workers.

The WHO health-system building blocks framework, while providing a transparent and policy-relevant structure for presenting the findings, is primarily descriptive and constrained the analysis toward categorization by system domain rather than toward theorization of the mechanisms linking governance, financing, workforce, and service-delivery barriers; future studies should consider applying implementation science frameworks such as CFIR, EPIS, or RE-AIM to examine these mechanisms, together with the political and institutional determinants of implementation, in greater depth. The analysis was further constrained by major evidence gaps within Somalia's oral health sector, including the absence of recent nationally representative oral health survey data, comprehensive mapping of public–private service providers, detailed workforce statistics, facility readiness assessments, and economic costing studies. Consequently, the proposed integration model should be regarded as a preliminary framework that requires validation through broader stakeholder consultation, district-level implementation research, potentially informed by implementation science frameworks such as CFIR, EPIS, or RE-AIM, economic evaluation, pilot testing, and meaningful engagement with communities and service users.

## Conclusion

Oral health integration into primary health care (PHC) in Somalia is necessary, feasible, and timely. Despite the substantial burden of untreated oral disease, oral health remains largely concentrated in the private sector, with limited availability in public PHC facilities. The findings reveal a persistent gap between policy recognition of oral health and its routine implementation within the health system. Stakeholders consistently identified weak governance and coordination, workforce shortages, inadequate infrastructure and supplies, limited regulation of private and informal providers, high out-of-pocket expenditure, workforce constraints, curative hospital-centered care, limited supplies, weak health information systems, and the absence of sustainable financing mechanisms as major barriers to integration.

At the same time, existing PHC reforms, EPHS implementation, and the broader pursuit of Universal Health Coverage (UHC) create important opportunities to integrate essential oral health services into the health system. The study suggests that the most practical and sustainable approach is a phased, health-system-embedded model of integration. Initial efforts should focus on strengthening governance, policy leadership, workforce capacity, service readiness, and referral systems. This foundation can support the progressive introduction of community- and PHC-based oral health promotion, prevention, screening, counselling, early recognition of oral diseases, and referral. As system capacity expands, complementary investments in regulation, commodities, financing mechanisms, routine DHIS2 monitoring, and essential curative services can be implemented incrementally.

Rather than establishing a separate vertical dental programme, integrating oral health within existing PHC and UHC structures offers a more realistic, equitable, and sustainable pathway for Somalia. By prioritizing prevention, early intervention, and system strengthening, Somalia can progressively improve access to essential oral health services and advance towards a more people-centred and inclusive health system. Future implementation should be supported by pilot testing, operational research, stakeholder engagement, and ongoing monitoring to ensure that integration strategies remain feasible, effective, and responsive to population needs.

## Data Availability

The original contributions presented in the study are included in the article/Supplementary Material, further inquiries can be directed to the corresponding author/s.
