# Supplementary material for: Integration of oral health into primary health care in Somalia: opportunities, barriers, and system-level implications for universal health coverage
Source: Front Oral Health. 2026 Oct 7;7:1930618. doi: 10.3389/froh.2026.1930618 (PMC13683106; doi:10.3389/froh.2026.1930618)
Supplement: Supplementary file 1 [file Datasheet1.pdf]

Key Informant Interview Guide

Integration of Oral Health into Primary Health Care in Somalia: Policy Positioning, System-Level Barriers, Opportunities, and Feasible Integration Pathways

Target Group 1/2: National Policy Makers

Head of the Oral Health Unit, Health-System Technical Advisers

(Introduce yourself and welcome the key informant to the interview. Ask if any further clarifications are needed regarding the study and related aspects before starting the interview)

1. Can you briefly describe your current role and responsibilities, how long you have been working in the health sector, and your experience related to primary health care or oral health services?
2. How is oral health currently prioritized within Somalia's health system, and to what extent is it included in national policies such as the EPHS or UHC plans?
3. Are there clear policies or guidelines supporting oral health integration into PHC?
4. What governance or leadership challenges affect oral health services?
5. How are oral health services currently financed in Somalia, what challenges exist in funding oral health within PHC, and are there opportunities to include oral health in publicly funded health services?
6. How would you describe the availability and distribution of oral health professionals, and what challenges exist in training, recruitment, and retention of the dental workforce?
7. What is your opinion on task-sharing (e.g., involving Female Health Workers in basic oral care)?
8. Are oral health services currently integrated into PHC facilities, what types of oral health services are available at primary care level, what are the major barriers to delivering oral health services through PHC, and how can oral health be integrated into existing services such as MCH?
9. Is oral health data routinely collected within health information systems (e.g., DHIS2), what challenges exist in monitoring oral health indicators, and how can data systems be improved to support oral health integration?
10. Are essential oral health supplies and equipment available at PHC facilities, and what challenges exist in procurement and supply of dental materials?
11. How would you describe community awareness and attitudes toward oral health, what barriers exist at the community level (e.g., cultural beliefs, affordability), and what role can community health workers play in improving oral health?
12. What opportunities exist for integrating oral health into PHC in Somalia, what strategies would you recommend to improve access to oral health services, and how can oral health be incorporated into national UHC and NCD strategies?
13. Is there anything else you would like to add regarding oral health services in Somalia, and do you have any recommendations for policymakers?

(Thank the participant for their time and contribution to the discussion)

Target Group 3: Health Professional Regulators

Representatives of the National Health Professionals Council (NHPC) responsible for professional regulation, registration, standards, and quality assurance

(Introduce yourself and welcome the key informant to the interview. Ask if any further clarifications are needed regarding the study and related aspects before starting the interview)

1. What is your current role and responsibilities within the National Health Professional Council? How long have you worked in health professional regulation, accreditation, or workforce development?
2. What is the role of the NHPC in professional licensing, accreditation of health training institutions, regulation of health professionals, and workforce standards and competency development?
3. How is oral health currently positioned within national health workforce policies and regulatory frameworks? Are there existing regulations, professional standards, or accreditation requirements for oral health practice?
4. What regulatory challenges affect the integration of oral health into PHC services? What policy or regulatory reforms are needed to strengthen this integration?
5. How effective is coordination between the Ministry of Health, NHPC, universities and training institutions, the Somali Dental Association, and development partners?
6. To what extent are oral health competencies included in the training of nurses, midwives, community health workers, and other PHC cadres? What opportunities exist for incorporating oral health into pre-service and in-service training curricula?
7. Should non-dental health workers be trained to provide basic oral health promotion, screening, and referral? What competency standards would be needed for task-sharing of basic oral health services within PHC?
8. How can universities and professional councils support oral health integration through education and accreditation?
9. How can workforce planning and task-sharing support the integration of oral health into PHC? Which PHC cadres are most suitable for delivering oral health education, screening, and referral, and what safeguards are needed to ensure quality and patient safety?
10. Does Somalia currently maintain reliable data on the oral health workforce? What gaps exist in this information, and how could workforce data systems be strengthened to support planning?
11. What are the major barriers to integrating oral health into PHC from a workforce and regulatory perspective? (Probe: workforce shortages, training gaps, regulatory constraints, limited standards, weak coordination). Which barriers are most significant at the policy, institutional, and workforce levels?
12. What opportunities exist to strengthen oral health integration through workforce reforms? How feasible is integration from a regulatory and workforce perspective, and what immediate, medium-, and long-term actions should be prioritized?
13. What role should the Ministry of Health, NHPC, universities, the Somali Dental Association, development partners, and other professional associations play in strengthening integration? What policy reforms or workforce investments would you recommend to policymakers? Is there anything else you would like to add?

(Thank the participant for their time and contribution to the discussion)

Target Group 4: Academics and Training Institutions

Deans, academic coordinators, and relevant faculty representatives involved in dental and oral health education, training, and research

(Introduce yourself and welcome the key informant to the interview. Ask if any further clarifications are needed regarding the study and related aspects before starting the interview)

1. What is your current role and responsibilities as Dean of the Faculty of Dentistry? How long have you worked in dental education and oral health systems?
2. What has been your involvement in oral health policy development, dental workforce training, public health or PHC initiatives, and community oral health programs?
3. How would you describe the current oral health situation in Somalia? What are the major challenges, which population groups are most affected, and how adequately is oral health addressed within the health system?
4. To what extent is oral health reflected in national health policies and strategic frameworks? How well is it integrated within PHC, UHC initiatives, the EPHS, and NCD programs?
5. What governance or policy gaps limit oral health integration? How effective is coordination among the Ministry of Health, universities, the Somali Dental Association, development partners, and private sector dental providers?
6. How adequate is the current dental workforce in Somalia, and what major workforce challenges affect oral health service delivery?
7. Does the current dental curriculum adequately prepare graduates for PHC-oriented oral health care? Should integration into PHC be strengthened within undergraduate and postgraduate training, and what competencies should non-dental health workers acquire?
8. How can universities contribute to task-sharing and workforce development for oral health?
9. What are the major research gaps in oral health in Somalia? How available is evidence for policy and planning, and what role should universities play in generating and using this evidence?
10. How feasible is integrating oral health into Somalia's PHC system? Which PHC platforms (community programs, schools, RMNCAH, nutrition, NCD clinics) offer the greatest opportunities, what basic services should be included, what referral mechanisms are needed, and what role can prevention play?
11. What are the major barriers to oral health integration in Somalia? (Probe: policy, financing, workforce, infrastructure, service delivery, awareness, data systems). Which barriers are most critical for successful integration?
12. What opportunities currently exist for integrating oral health into PHC? Are there successful models from Somalia or other countries to adapt, how can universities support implementation, and what role can professional associations play?
13. What immediate, medium-term, and long-term actions should be prioritized to strengthen oral health integration? What recommendations would you give to the Ministry of Health, universities, the Somali Dental Association, development partners, and training institutions? Is there anything else you would like to add?

(Thank the participant for their time and contribution to the discussion)

Target Group 5: Dental Professional Association

Representatives of the Somalia Dental Professional Association involved in professional development, advocacy, standards of practice, and oral health policy

(Introduce yourself and welcome the key informant to the interview. Ask if any further clarifications are needed regarding the study and related aspects before starting the interview)

1. What is your current position and responsibilities within the Dental Professional Association? How long have you been involved in the dental profession and/or the Association? What are the Association's main roles in Somalia, and how is it currently involved in oral health policy, professional development, advocacy, or service improvement?
2. From the Association's perspective, what is the current status of oral health and oral health services in Somalia? What are the most common oral health problems, how accessible and affordable are essential services, what are the major gaps in availability, quality, and distribution, and how much emphasis is placed on prevention versus treatment?
3. How is oral health currently positioned within Somalia's national health policies and strategies? Are existing policies and guidelines adequate to support oral health service delivery and integration into PHC, and why or why not? What are the main policy and governance gaps?
4. How effective is coordination between the Association and key stakeholders (Ministry of Health, NHPC/regulatory bodies, universities, public and private facilities, NGOs and development partners, PHC/community programs)? What role should the Association play in policy development, implementation, and monitoring, and what policy reforms are most important?
5. How would you describe the current regulation and professional standards governing dental practice in Somalia? Are there adequate clinical guidelines, standards, or protocols, and what challenges affect quality and safety across public and private facilities? What role does or could the Association play in promoting standards, continuing professional development, ethics, quality improvement, and patient safety, and what additional measures are needed?
6. How would you describe the current oral health workforce in terms of numbers, skills, distribution, and competencies? What are the major workforce gaps and challenges in training, recruitment, deployment, and retention? Are current training programs adequately preparing professionals for PHC priorities, what role could the Association play in strengthening continuing professional development, and how could non-dental PHC workers be trained to provide basic oral health promotion, screening, and referral?
7. What are the main financing challenges affecting oral health services in Somalia? To what extent does out-of-pocket payment affect access, which population groups face the greatest barriers, and should essential oral health interventions be incorporated into the EPHS and future UHC benefit packages?
8. How well is oral health currently integrated into PHC services in Somalia? Which essential interventions could realistically be delivered at PHC level, how could oral health be integrated into existing programs (community health, school health, RMNCAH, nutrition, NCD), what roles should dentists play in supporting PHC-level services, and what model of integration would be most feasible?
9. How adequate is the current availability of oral health data in Somalia? What are the major gaps in surveillance, research, and reporting, what indicators should be added to the national health information system, and how could stakeholders collaborate to strengthen research and evidence generation?
10. What are the major challenges related to dental infrastructure, equipment, materials, medicines, and supplies? How do these affect service availability and quality, what challenges exist in procurement and supply-chain management, and what minimum equipment and infrastructure would be needed at PHC level?
11. What are the main barriers to integrating oral health into PHC in Somalia? (Probes: policy prioritization, governance, financing, workforce, professional/regulatory challenges, infrastructure, referral systems, data, public awareness, geographic inequality). Which barriers are most critical, and which could be addressed in the short term?

12. What opportunities currently exist for strengthening oral health integration into PHC? Which programs or platforms offer the best opportunities, what partnerships are necessary, how could the Association contribute (advocacy, workforce development, standards, awareness, research, quality improvement, implementation), and what would facilitate sustainable scale-up?
13. How feasible is integrating essential oral health services into PHC, considering Somalia's current health-system capacity? Which interventions should be prioritized first, what practical actions could happen in the next 1–2 years, and what medium- and long-term investments and commitments are needed from key stakeholders? What are your top three recommendations, what should be the single most important immediate action for policymakers, what role should the Association take, and is there anything else you would like to add?

(Thank the participant for their time and contribution to the discussion)

Target Group 6/7/8: Oral Health Service Providers

Representatives from public hospitals, private dental practitioners, and social-enterprise providers involved in the delivery of oral health and dental services

(Introduce yourself and welcome the key informant to the interview. Ask if any further clarifications are needed regarding the study and related aspects before starting the interview)

1. What is your current position and responsibilities within the hospital? How long have you worked in hospital management and leadership, and what role does the hospital play in providing primary health care and referral services?
2. How would you describe the current status of oral health services in your hospital (preventive services, screening, dental treatment, emergency care, referral)? What are the most common oral health problems seen among patients, and to what extent are services linked with other hospital departments and PHC facilities?
3. How is oral health prioritized within your hospital? What leadership or governance challenges affect service delivery, how effective is coordination with the Ministry of Health, other hospitals, PHC facilities, universities, NGOs/development partners, and the Somali Dental Association, and what policy changes would help strengthen services?
4. How are oral health services financed within your hospital? What financial challenges affect delivery, are services affordable for most patients, and should oral health be included in the EPHS and UHC benefit package, and why?
5. Does your hospital have adequate oral health personnel (dentists, dental therapists, dental nurses, dental technicians)? What workforce gaps exist, what challenges affect recruitment and retention, and how can non-dental health workers contribute to oral health promotion and early detection?
6. What are the major gaps in oral health service delivery within your hospital? How effective are referral systems between PHC facilities and hospitals, and how could services be better integrated into outpatient care, community health, school health, RMNCAH, nutrition, and NCD programs?
7. What practical integration model would be most feasible in your hospital?
8. Is oral health data routinely collected in your hospital? What indicators are reported, what challenges affect data collection and use, and what improvements are needed?
9. Does your hospital have adequate infrastructure for oral health services? Are equipment, materials, and supplies consistently available, what challenges affect procurement and supply chain management, and what investments are most urgently needed?
10. What are the main barriers to integrating oral health into PHC services in Somalia? (Probe: policy, financing, workforce, infrastructure, referral systems, public awareness). Which barriers are most significant at the national, hospital, and community levels?
11. What opportunities exist for integrating oral health into PHC services? Which existing programs (community health, school health, RMNCAH, nutrition, NCD) could support integration, and what factors would facilitate implementation?
12. How feasible is oral health integration into PHC in Somalia? What practical strategies should be prioritized in the short term, and what medium- and long-term investments are needed?
13. What are your top three recommendations for improving oral health services in Somalia? What immediate actions should policymakers prioritize, and is there anything else you would like to add?

(Thank the participant for their time and contribution to the discussion)
