# Supplementary material for: Integration of oral health into primary health care in Somalia: opportunities, barriers, and system-level implications for universal health coverage
Source: Front Oral Health. 2026 Oct 7;7:1930618. doi: 10.3389/froh.2026.1930618 (PMC13683106; doi:10.3389/froh.2026.1930618)
Supplement: Supplementary file 2 [file Supplementaryfile1.docx]

## Supplementary Table S1. Coding framework

Supplementary Table S1. Coding framework used for thematic analysis

| **Code** | **Operational definition** | **Example indicators in transcripts** | **WHO mapping** |
| --- | --- | --- | --- |
| GOV_POLICY_IMPLEMENTATION_GAP | Statements describing oral health as included in EPHS/HSSP/UHC but not implemented, funded, or operationalized. | EPHS but not implemented; policy on paper; under-prioritized. | Leadership/governance |
| GOV_REGULATION_COORDINATION | Statements about weak coordination, lack of standards, unlicensed practice, accreditation, licensing, or regulation of private providers/training institutions. | Weak coordination; lack of regulations; need MoH/NHPC/SDA roles. | Leadership/governance |
| FIN_OOP_AFFORDABILITY | Statements about OOP payment, lack of insurance, cost barriers, private financing, or delayed care due to cost. | Private only; high OOP; no public insurance; affordability challenge. | Financing |
| WFD_SHORTAGE_MALDISTRIBUTION | Statements about shortage, uneven distribution, absence of allied cadres, or lack of specialists. | Rural shortage; lack of dental hygienists/nurses/therapists; stretched hospitals. | Health workforce |
| WFD_TRAINING_TASKSHARING | Statements about PHC worker competencies, curricula, task-sharing, CHWs, nurses, midwives, CPD, or practical training. | Train CHWs/nurses; curriculum too hospital-centred; need competencies. | Health workforce |
| SD_CURATIVE_URBAN_HOSPITAL | Statements about curative, emergency-driven, urban, hospital-centred care and delayed presentation. | Advanced caries; abscess; tooth loss; tertiary bottlenecks. | Service delivery |
| SD_MCH_PHC_ENTRYPOINTS | Statements about ANC, child health, nutrition, immunization, school health, NCDs or community platforms as entry points. | Screening in ANC; school health; immunization; nutrition contacts. | Service delivery / PHC |
| HIS_DATA_INVISIBILITY | Statements about DHIS2, facility records, absent indicators, workforce data, research gaps or surveillance. | No DHIS2 oral health indicators; EMR not linked; no national survey. | Health information systems |
| COM_INFRA_SUPPLY | Statements about dental equipment, consumables, sterilization, electricity, water, import delays, counterfeit materials or maintenance. | No dental rooms; imported supplies; equipment maintenance; IPC gaps. | Medical products/technologies |
| INT_FEASIBLE_PHASED_MODEL | Statements proposing feasible integration steps, phased implementation, minimum package, referral, regulation, financing, or policy roadmap. | Roadmap; minimum package; referral pathway; CHW training; phased scale-up. | Cross-cutting |

Table 7. Feasible integration pathways for Somalia

| **Pathway** | **Entry point** | **Core intervention package** | **Lead actors** |
| --- | --- | --- | --- |
| Policy | HSSP, EPHS, PHC, NCD and UHC reforms | Oral health focal point; roadmap; EPHS oral health module; standards; regulation | Federal Ministry of Health, state ministries, NHPC, SDA, partners |
| PHC service delivery | Health posts, health centres, outpatient departments | Education, screening, pain triage, infection danger-sign recognition, referral | PHC clinicians, nurses, midwives |
| Community health | Household visits, outreach, campaigns | Caregiver counselling, sugar-risk reduction, fluoride toothpaste promotion, danger-sign referral | CHWs/Marwo Caafimaad, community supervisors |
| School health | Schools and child/adolescent platforms | Oral hygiene education, sugar-risk reduction, screening and referral | Ministries of Health and Education, teachers, dental schools |
| NCD and nutrition | Diabetes, tobacco, cancer-awareness, growth monitoring, IYCF, malnutrition services | Shared risk-factor counselling, periodontal-health awareness, oral cancer warning signs, feeding-difficulty referral | NCD, nutrition and PHC teams |
| RMNCAH | ANC, PNC, immunization, child and adolescent health | Maternal oral health counselling; early childhood caries prevention; adolescent risk counselling | Midwives, nurses, RMNCAH teams |
| Workforce | Pre-service and in-service training | Modular competencies, task-sharing, referral skills, IPC and patient safety | Training institutions, MoH, NHPC, universities |
| Financing and commodities | EPHS costing, partner alignment, supply chain | Finance prevention first; later subsidize emergency care; include staged supplies and IPC materials | MoH, donors, NGOs, supply-chain units |
| HMIS | DHIS2/HMIS and supervision | Minimal indicators for screening, counselling, referrals and readiness | HMIS units, district teams |

Table 8. Proposed minimum oral health package by level of care

| **Level** | **Immediate services** | **Later services when ready** | **Referral criteria** |
| --- | --- | --- | --- |
| Community | Oral health education; child toothbrushing counselling; sugar-risk reduction; early care-seeking messages | School and community fluoride-toothpaste promotion | Facial swelling, severe pain, trauma, feeding difficulty, persistent ulcer, suspicious lesion |
| Health post / PHC | Brief screening; oral hygiene and dietary counselling; pain assessment; danger-sign recognition; referral | Fluoride varnish in selected platforms if protocols and commodities are available | Abscess, trauma, persistent pain, suspected malignancy, spreading infection |
| Health centre | Screening, counselling, analgesia, infection triage, referral | Simple extractions or atraumatic restorative treatment only where trained staff, sterilization and supplies exist | Complex infection, trauma, uncontrolled bleeding, medically complex patients |
| District/referral facility | Emergency dental care, extractions, infection management, trauma care | Basic restorative and periodontal care | Malignancy, maxillofacial trauma, complex surgical cases |
| National/tertiary | Specialist consultation, oral cancer diagnostic pathway, complex trauma care | Training hub, research, quality assurance | Advanced oncology, maxillofacial, reconstructive, periodontal and prosthetic care |

Table 9. Proposed minimal HMIS/DHIS2 oral health indicator set

| **Indicator** | **Numerator** | **Denominator** | **Reporting level** | **Frequency** |
| --- | --- | --- | --- | --- |
| PHC clients screened for oral symptoms | Clients screened for oral pain, swelling, trauma or oral lesions | Total eligible outpatient clients | Facility | Monthly |
| Children/caregivers counselled | Children or caregivers counselled on toothbrushing, fluoride toothpaste and sugar reduction | Total child health or nutrition contacts | Facility/community | Monthly |
| Pregnant women counselled | ANC clients receiving oral health counselling | Total first ANC visits | Facility | Monthly |
| Urgent oral referrals | Clients referred for severe pain, swelling, trauma, bleeding or infection | Total clients screened | Facility | Monthly |
| Suspected oral cancer referrals | Clients referred for persistent ulcer, suspicious lesion or unexplained oral bleeding | Total clients screened | Facility/referral | Monthly/quarterly |
| Schools reached | Schools receiving oral health education or screening | Schools in catchment area | District | Quarterly |
| Basic oral health readiness | Facilities with counselling materials, torch, gloves, tongue depressors, referral forms and analgesics | Total reporting PHC facilities | District/facility | Quarterly |
