# Supplementary material for: Integration of oral health into primary health care in Somalia: opportunities, barriers, and system-level implications for universal health coverage
Source: Front Oral Health. 2026 Oct 7;7:1930618. doi: 10.3389/froh.2026.1930618 (PMC13683106; doi:10.3389/froh.2026.1930618)
Supplement: Supplementary file 3 [file Supplementaryfile2.docx]

| **Document** | **Issuing body** | **Year** | **Type** |
| --- | --- | --- | --- |
| Somalia Health Sector Strategic Plan III (HSSP III) 2022–2026 | Federal Government of Somalia, Ministry of Health | 2021 | National Health strategic plan |
| Essential Package of Health Services (EPHS) 2020 | Federal Republic of Somalia, Ministry of Health | 2021 | National health service package |
| Investment Case for the Somali Health Sector 2022–2027 | Federal Republic of Somalia, Ministry of Health | 2022 | National investment strategy |
| Somali Community Health Strategy: Health Services at the Doorstep of Somali Communities | Federal Government of Somalia, Ministry of Health | 2015 | National community health strategy |
| The ’Marwo Caafimaad’ Programme: Compendium to implement Community-based Female Health Workers’ Programme | World Health Organization, Somalia, UNICEF Somalia and UNFPA Somalia together with Ministry of Health, Central South Somalia | 2014 | National community health strategy |
| Somalia Community Health Strategy 2023–2027, | Federal Government of Somalia, Ministry of Health | 2026 | National community health strategy |
| Oral Health Country Profile: Somalia | World Health Organization | 2022 | International country profile |
| Global Oral Health Status Report | World Health Organization | 2022 | International technical report |
| Global Strategy and Action Plan on Oral Health 2023–2030 | World Health Organization | 2024 | International strategic guidance |
